# Structural properties of Sb_2_S_3_ under pressure: evidence of an electronic topological transition

**DOI:** 10.1038/srep24246

**Published:** 2016-04-06

**Authors:** Ilias Efthimiopoulos, Cienna Buchan, Yuejian Wang

**Affiliations:** 1 Department of Physics, Oakland University, Rochester, MI, 48309, USA

## Abstract

High-pressure Raman spectroscopy and x-ray diffraction of Sb_2_S_3_ up to 53 GPa reveals two phase transitions at 5 GPa and 15 GPa. The first transition is evidenced by noticeable compressibility changes in distinct Raman-active modes, in the lattice parameter axial ratios, the unit cell volume, as well as in specific interatomic bond lengths and bond angles. By taking into account relevant results from the literature, we assign these effects to a second-order isostructural transition arising from an electronic topological transition in Sb_2_S_3_ near 5 GPa. Close comparison between Sb_2_S_3_ and Sb_2_Se_3_ up to 10 GPa reveals a slightly diverse structural behavior for these two compounds after the isostructural transition pressure. This structural diversity appears to account for the different pressure-induced electronic behavior of Sb_2_S_3_ and Sb_2_Se_3_ up to 10 GPa, i.e. the absence of an insulator-metal transition in Sb_2_S_3_ up to that pressure. Finally, the second high-pressure modification appearing above 15 GPa appears to trigger a structural disorder at ~20 GPa; full decompression from 53 GPa leads to the recovery of an amorphous state.

The Sb_2_S_3_ material (stibnite) is a well-known binary semiconductor with an optical band gap *E*_g _~ 1.7 eV^1^. This material constitutes a promising candidate for solar energy conversion^2^ and optoelectronic applications^3^. At ambient conditions, Sb_2_S_3_ crystallizes in a complex orthorhombic structure (SG *Pnma*, *Z* = 4, U_2_S_3_-type)^4^. This *Pnma* phase can be described as a layered structure, consisting of parallel molecular (Sb_4_S_6_)_n_ ribbon-like chains along the short *b*-axis held together by weak intermolecular forces. The Sb^3+^ ions are located at two different sites in this phase, and their coordination environment can be described as sevenfold for the Sb(1) ion and eightfold (7 + 1) for the Sb(2) ion, respectively (Fig. 1). The same structure is adopted by Sb_2_Se_3_^5^, whereas Sb_2_Te_3_ adopts a rhombohedral structure (SG 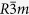, *Z* = 3) made up of SbTe_6_ octahedral layers piled up along *c*-axis^6^ due to the absence of the Sb^3+^ lone electron pair stereochemical activity^7^.

Very recently, Sb_2_Se_3_ was shown also to undergo an electronic topological transition (ETT) near 3 GPa^8^, with theoretical works corroborating such behavior^9^,^10^. This ETT, which appears to be a common trend for these systems^11^, was manifested as a second-order isostructural transition via compressibility changes in several structural parameters^11^,^12^. We remind here that an ETT occurs when a band extremum, which is associated to a Van Hove singularity, crosses the Fermi energy (*E*_F_) and leads to a strong redistribution of the electronic density of states (EDOS) near *E*_F_. This EDOS redistribution induces a second-order isostructural transition, i.e. a transition without any volume discontinuity at the transition point or changes in the crystalline symmetry. The elastic constants are affected by the ETT, however, hence leading to distinct compressibility changes of the material under study^13^,^14^. In addition, high-pressure resistivity measurements of Sb_2_Se_3_ revealed also an insulator-metal transition near 3 GPa, whereas pressure-induced superconductivity was observed above 10 GPa^12^. The superconducting state persisted up to 40 GPa, with further compression leading to the transformation of the *Pnma* structure into a disordered body-centered cubic (bcc) phase above 55 GPa^15^.

A similar ETT was recently observed for Sb_2_S_3_ by means of Raman spectroscopic and resistivity probes near 5 GPa, with a second phase transition following at 20 GPa^16^. Even though a x-ray diffraction (XRD) study of Sb_2_S_3_ up to 10 GPa showed the persistence of the *Pnma* phase up to that pressure^17^, the mechanism of ETT at 5 GPa, the detection of the second transition at 20 GPa, as well as the possibility of additional structural modifications upon further compression^15^,^18^,^19^,^20^, calls for an updated structural investigation of Sb_2_S_3_ in a more extended pressure range.

We present here our combined high-pressure Raman and XRD studies on Sb_2_S_3_ up to 25 GPa and 53 GPa, respectively. Overall, we have detected two phase transitions at 5 GPa and 15 GPa. The first transition is manifested via compressibility changes in several structural parameters, an observation which correlates strongly with the reported ETT^16^. As for the second transition at 15 GPa, this new phase could not be identified due to the onset of structural disorder above 20 GPa. Full decompression of Sb_2_S_3_ from 53 GPa leads to the recovery of an amorphous state.

## Results

Given that Raman spectroscopy is a more sensitive probe for isostructural transitions^11^, we present first our high-pressure Raman spectroscopic investigation on Sb_2_S_3_. According to group theory, a sum of thirty Raman-active modes are expected for the *Pnma* phase of Sb_2_S_3_^21^:


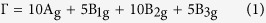


We can resolve ten broad bands in our Raman spectra (Fig. 2), which is consistent with the reported literature^16^,^22^,^23^. The mode assignment is adopted from the polarization studies of Sereni *et al*.^23^ (Table 1). Upon increasing pressure, most of the Raman modes upshift in frequency; on the contrary, the high-frequency A_g_(4), A_g_(5), and B_1g_(5) modes downshift in energy up to 5 GPa. Beyond that pressure, the A_g_(4) and B_1g_(5) features display a compressibility change, with the A_g_(4) mode exhibiting a completely different pressure-induced behavior after 5 GPa with a positive pressure slope [Fig. 2(b) and Table 1]. In addition, the pressure slope of the B_1g_(5) mode reduces substantially beyond 5 GPa. As for the A_g_(5) mode, it merges with its neighboring B_1g_(5) mode at 7 GPa, and could not be followed above that pressure. Since these modes correspond to the stretching vibrations of the shorter Sb-S distances^22^, the observed compressibility changes should reflect the pressure-induced behavior of the corresponding bond lengths. Overall, our observations are in excellent agreement with the recent study of Sorb *et al*.^16^.

Further compression of Sb_2_S_3_ results in the appearance of two new low-intensity features beyond 15 GPa [denoted as M1 and M2 in Fig. 2(b), see also supplementary Fig. S1]. Since the strongest Raman features of elemental Sb^24^ and S^25^,^26^,^27^ do not reside in these frequencies, we can safely exclude any decomposition and attribute the appearance of the M1 and M2 features to a pressure-induced phase transition. The M2 feature was also detected in the study of Sorb *et al*. above 20 GPa^16^. Beyond 15 GPa, however, the Raman modes exhibit pronounced broadening; consequently, the Raman spectra become rather featureless at 22 GPa [Fig. 2(a)]. Full decompression from 25 GPa leads to the recovery of the original Sb_2_S_3_ Raman spectrum, indicating the reversibility of the pressure-induced Raman changes.

Having documented the pressure-induced changes from our Raman investigation, we now focus on the structural properties of Sb_2_S_3_ under pressure. Selected XRD patterns are presented in Fig. 3. As we can observe, the Bragg peaks of the *Pnma* phase could be detected up to the highest pressure, i.e. 53 GPa. The Bragg peaks, however, exhibit significant broadening beyond 20 GPa, indicating the onset of structural disorder above that pressure. This pressure-induced structural disorder is most likely responsible for the featureless Raman spectra at 22 GPa [Fig. 2(a)]. Further compression leads to the substantial broadening of the *Pnma* Bragg peaks in our XRD patterns. Upon decompression from 53 GPa we obtain an amorphous-like state, in apparent contradiction with our Raman study where the original phase is recovered upon decompression from 25 GPa [Fig. 2(a)]. It appears that unloading from a significantly larger pressure favors the quenching of an amorphous state instead of the original structure.

In addition, a new Bragg peak could be detected at 2θ ≈ 6.6° near 15 GPa (Fig. 3 and supplementary Fig. S2). This feature could not be assigned either to the *Pnma* or any “contaminating” phase such as rhenium (gasket material), helium, or ruby. Therefore, the appearance of this extra Bragg peak signals a structural transition of Sb_2_S_3_ at 15 GPa, in excellent agreement with our Raman study (Fig. 2). Attempts to fit the XRD patterns with any of the reported high-pressure phases of related A_2_B_3_ compounds^18^,^19^,^20^,^28^ proved unsuccessful. A possible elemental decomposition into Sb^29^ and S^30^ could also not account for this novel peak. Therefore, we speculate that the Sb_2_S_3_ XRD patterns consist of a mixture of two phases above 15 GPa, i.e. the *Pnma* structure and a high-pressure modification. Due to the pronounced Bragg peak broadening and the fact that this high-pressure phase is characterized by a single Bragg feature, however, its identification becomes unattainable at this point.

Given the aforementioned Bragg peak overlap and broadening upon pressure increase, the interatomic and lattice parameters of the *Pnma* phase could be obtained reliably up to 9 GPa and 20 GPa, respectively. All of these structural data are provided in supplementary Tables S1 and S2, whereas the extracted *P*-*V* data are shown in Fig. 4. As we can observe, there is a clear compressibility change in the volume and in the orthorhombic axial ratios near 5 GPa, in excellent agreement with the compressibility changes observed in our Raman spectra (Fig. 2). By taking into account the pressure-induced behavior of isostructural Sb_2_Se_3_^8^,^12^ and Bi_2_S_3_^31^ compounds, and in close comparison with the related Bi_2_Te_3_^32^,^33^, we attribute these compressibility changes to an *isostructural* transition of Sb_2_S_3_ near 5 GPa. This isostructural transition is most likely the signature of the reported ETT in Sb_2_S_3_^16^, reflecting a change in the topology of the Fermi surface^8^,^10^,^11^,^13^.

The fitting of the *P*-*V* data to a Birch-Murnaghan Equation of State (B-M EoS) yielded volumes and bulk moduli values of *V*_0_ = 488.2 (4) Å^3^ and *B*_0_ = 27.2(6) GPa, and *V* = 434.2(7) Å^3^ and B = 65(2) GPa (calculated at *P* = 5 GPa) before and after the isostructural transition, with fixed bulk moduli derivatives of *B*′_0_ = 6 and *B*’ = 4, respectively. The *B*_0_ value of Sb_2_S_3_ prior to the transition is consistent with that of Lundegaard *et al*.^17^ and in line with the *B*_0_ value of Sb_2_Se_3_^12^,^15^.

Except from these compressibility changes in the lattice parameters and the bulk volume, inspection of the interatomic parameters also reveals distinct changes above 4 GPa (Fig. 5). For example, we can observe that the Sb(1)-S(3) short bond distance *elongates* up to 4 GPa, whereas a reduction is evidenced above that pressure. Actually, this pressure-induced trend is in excellent agreement with the behavior of the stretching A_g_(4) Raman-active mode [Fig. 2(b)]. A similar trend applies for the S(3)-Sb(2)-S(3) bond angle (Fig. 5), which reflects the tiltings and distortions of the Sb(1)S_7_ polyhedra^12^. Overall, the isostructural transition can be readily witnessed from the behavior of the interatomic parameters, a common trend for these systems^12^,^15^,^31^.

## Discussion

Having resolved the structural evolution of Sb_2_S_3_ under pressure, a comparison between the pressure-induced behavior of Sb_2_S_3_ and the isostructural Sb_2_Se_3_ is in order. Both of these compounds exhibit isostructural transitions at the same pressure, i.e. close to 5 GPa^12^,^15^. In both cases, an electronic topological transition was put forward in order to account for the structural and vibrational changes beyond that pressure^8^,^12^,^16^. Interestingly, the high-pressure resistivity studies of these compounds reveal diverse behavior^12^,^16^. In particular, the room temperature resistivity of Sb_2_S_3_ was found to *increase* up to 5 GPa, where it reached saturation; two anomalies were also detected at 1.4 GPa and 2.4 GPa^16^. For Sb_2_Se_3_ on the other hand, a reduction of resistivity was observed up to 3.5 GPa, where an *insulator-metal* transition takes place; further compression leads to the induction of superconductivity at 10 GPa (*T*_c_ = 2 K)^12^. This pressure-induced behavior of Sb_2_Se_3_ resembles the transport properties of Bi_2_Se_3_, Bi_2_Te_3_, and Sb_2_Te_3_ compounds^34^,^35^,^36^,^37^,^38^,^39^. Given the fact that Sb_2_S_3_ and Sb_2_Se_3_ exhibit almost identical electronic band structures^7^,^40^, this diverse behavior in resistivity is puzzling.

**Figure 1:**
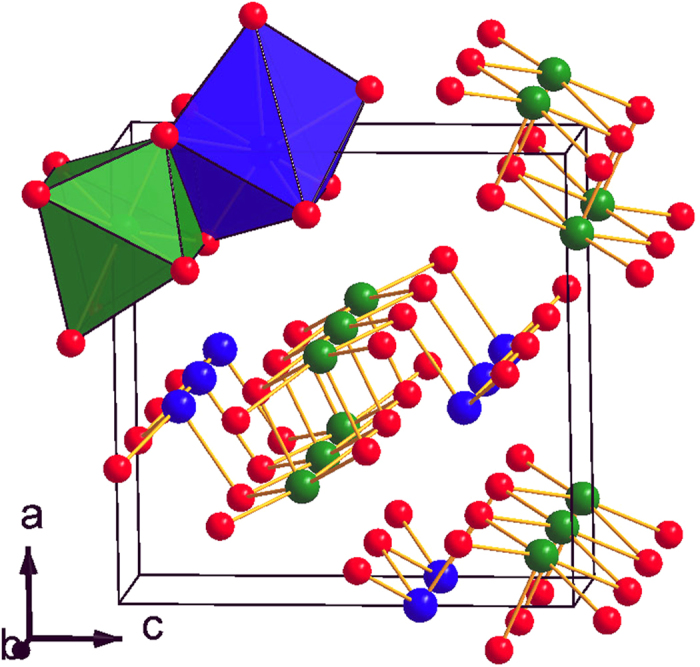
Unit cell of Sb_2_S_3_ at ambient conditions (SG *Pnma*, *Z* = 4). The blue, green, and red spheres correspond to the Sb(1), Sb(2), and S ions, respectively. The blue Sb(1)S_7_ and green Sb(2)S_7+1_ polyhedra are also displayed.

**Figure 2:**
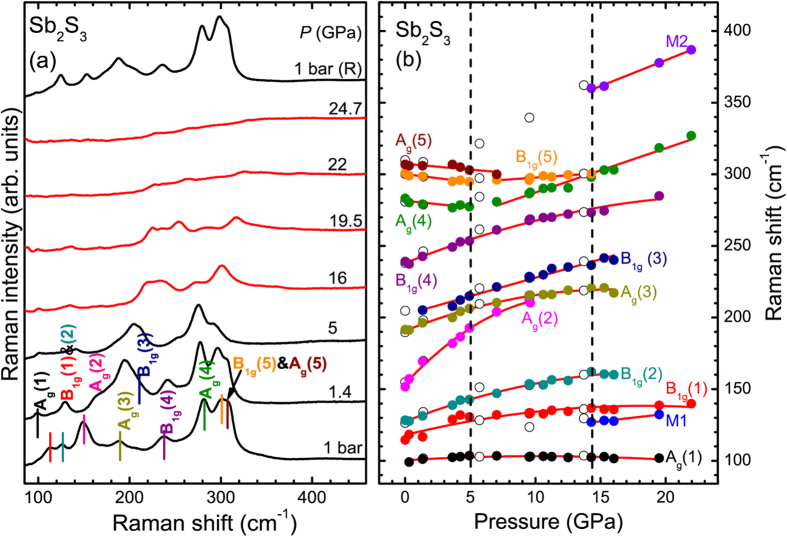
High-pressure Raman spectroscopic results of Sb_2_S_3_. (**a**) Raman spectra of Sb_2_S_3_ at selected pressures (*λ* = 532 nm, *T* = 300 K). Vertical lines indicate the Sb_2_S_3_ Raman-active modes. (**b**) Raman mode frequency evolution of Sb_2_S_3_ against pressure. Solid and open circles correspond to data collected upon compression and decompression, respectively. Solid lines represent least square fits. The dashed lines mark the onset of phase transitions (see text).

A direct comparison between the pressure-induced behavior of the Sb_2_S_3_ and Sb_2_Se_3_ structural parameters provides some hints. Generally, the compression mechanism for both compounds is practically identical up to 5 GPa^15^. As expected, the *b*-axis is the least compressible direction, since it contains the Sb_4_S(Se)_6_ molecular ribbons comprising the *Pnma* structure (Fig. 1). On the other hand, the *a*-axis and *c*-axis reduce much faster compared to *b*-axis under compression, with *a*-axis being more compressible than *c*-axis up to 5 GPa. This is easily evidenced from the increasing rate of the *c*/*a* axial ratio up to that pressure [Fig. 6(a)].

Beyond 5 GPa, however, Sb_2_S_3_ and Sb_2_Se_3_ exhibit slghtly diverse compressibility trends. In particular, the orthorhombic *c*-axis becomes more compressible and the *b*-axis less compressible in Sb_2_S_3_ than Sb_2_Se_3_ above 5 GPa; the *a*-axis exhibits similar compressibility for both compounds. As a result, the axial ratios of Sb_2_S_3_ behave differently than those of Sb_2_Se_3_ above 5 GPa, e.g. the Sb_2_S_3_ *c*/*a* axial ratio shows a more prominent decreasing trend beyond 5 GPa compared to the Sb_2_Se_3_ *c*/*a* axial ratio [Fig. 6(a)].

A closer look at the microscopic structural behavior reveals additional information. In Fig. 6(b,c) we plot the pressure-induced evolution of the X(3)-Sb(2)-X(3) bond angle, the volume of the Sb(1)X_7_ polyhedra (X = S, Se), and the Sb(1) cation eccentricity for both Sb_2_S_3_ and Sb_2_Se_3_ compounds. We note that the X(3)-Sb(2)-X(3) bond angle reflects the tiltings of the Sb(1)X_7_ polyhedra parallel to the *ac* plane^12^, whereas the cation eccentricity is a quantitative measure of the stereochemical activity of the Sb^3+^ lone electron pair (LEP); the larger the eccentricity value, the more active the LEP^17^,^41^. As we can observe, the X(3)-Sb(2)-X3 bond angles behave similarly for both Sb_2_S_3_ and Sb_2_Se_3_ up to 5 GPa, i.e. up to the isostructural transition [Fig. 6(b)]. Further compression, however, leads to the reduction of the S(3)-Sb(2)-S(3) bond angle in Sb_2_S_3_ in a more pronounced rate compared to the Se(3)-Sb(2)-Se(3) bond angle of Sb_2_Se_3_. Given that the LEPs are located at the *ac* plane^42^,^43^, this diverse bond angle changes mirror different Sb^3+^ LEP behaviors in these materials.

Indeed, in Fig. 6(c) we can observe that the Sb(1) cation eccentricities of Sb_2_S_3_ and Sb_2_Se_3_ display different behavior beyond 5 GPa. More precisely, the larger Sb(1) eccentricity value for Sb_2_S_3_ compared to Sb_2_Se_3_ indicates a larger stereochemical activity for the former after the isostructural transition. In other words, the Sb(1) LEP of Sb_2_S_3_ does not hybridize strongly with its neighboring S-p orbitals up to 10 GPa, as opposed to Sb_2_Se_3_ where the Sb(1) cation eccentricity value approaches an almost zero value after 5 GPa (almost complete orbital overlap). We speculate that this lack of LEP hybridization in Sb_2_S_3_ up to 10 GPa is the reason behind the different pressure-induced behavior in the electronic properties of Sb_2_S_3_ and Sb_2_Se_3_, i.e. the reason why an insulator-metal transition is observed for Sb_2_Se_3_^12^ and not in Sb_2_S_3_ (at least up to 10 GPa)^16^. Theoretical calculations are required, however, to verify this scenario.

**Figure 3:**
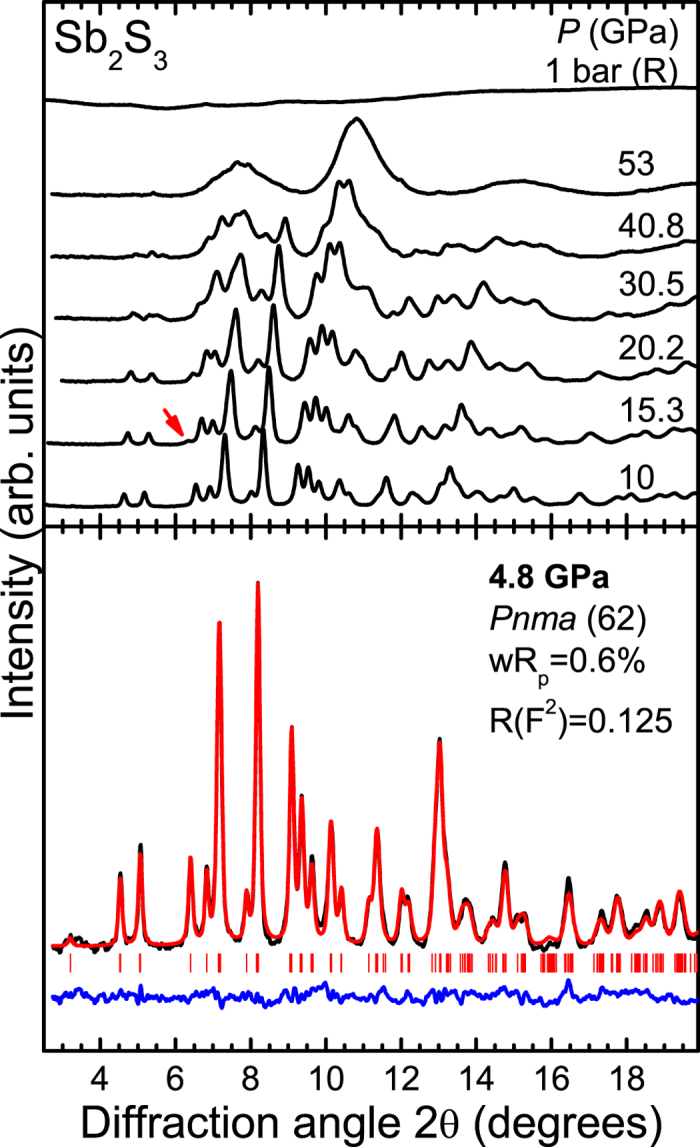
Selected XRD patterns of Sb_2_S_3_ at various pressures pressures (*λ* = 0.4246 Å, *T* = 300 K). The red arrow marks the new Bragg feature. An example of a Rietveld refinement at 4.8 GPa is also provided. The black and red solid lines correspond to the measured and the fitted spectra, whereas their difference is depicted as a blue line. Vertical ticks mark the Bragg peak positions for the *Pnma* phase.

**Figure 4:**
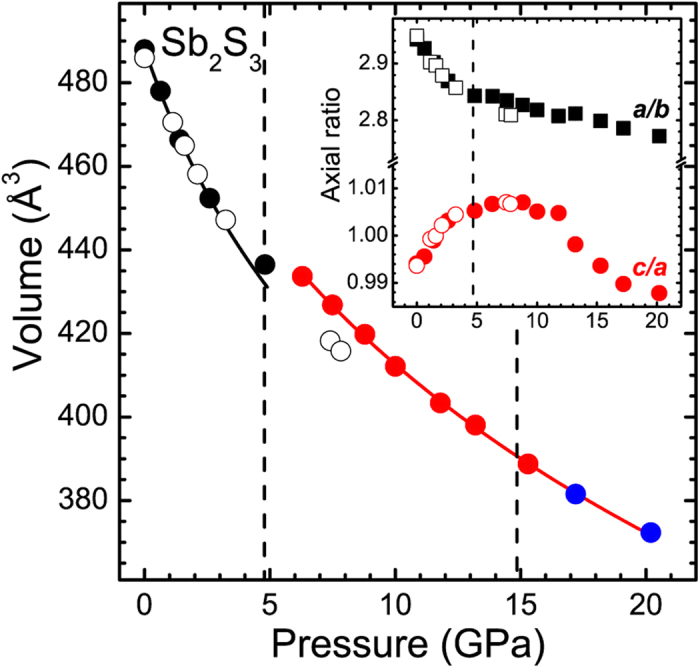
Plot of the unit cell volume as a function of pressure for the *Pnma* phase of Sb_2_S_3_. The solid lines represent the fitted Birch-Murnaghan Equation of State. The orthorhombic axial ratios are shown in the inset. The vertical dashed lines mark the onset of phase transitions (see text). The open symbols correspond to data from Lundegaard *et al*.^17^.

**Figure 5:**
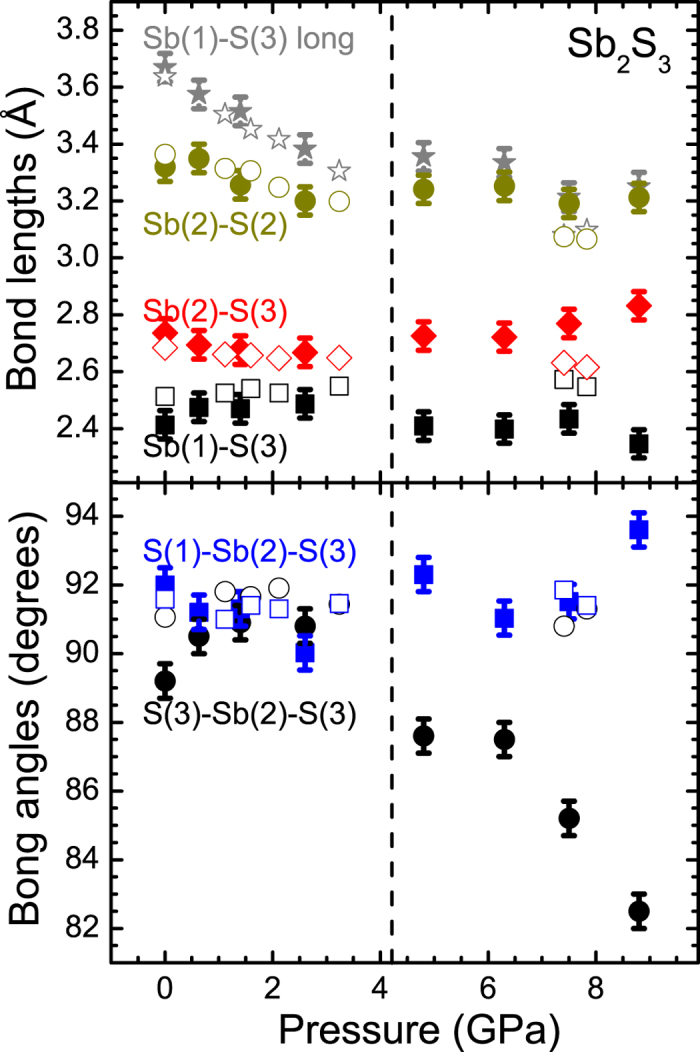
Distinct interatomic Sb_2_S_3_ parameters as a function of pressure. (**a**) Selected Sb-S bond lengths and (**b**) S-Sb-S bond angles up to 9 GPa. The vertical dashed line marks the onset of the isostructural transition. The nomenclature of the Sb and S ions is provided in supplementary Fig. S3. The open symbols correspond to data from Lundegaard *et al*.^17^.

**Figure 6:**
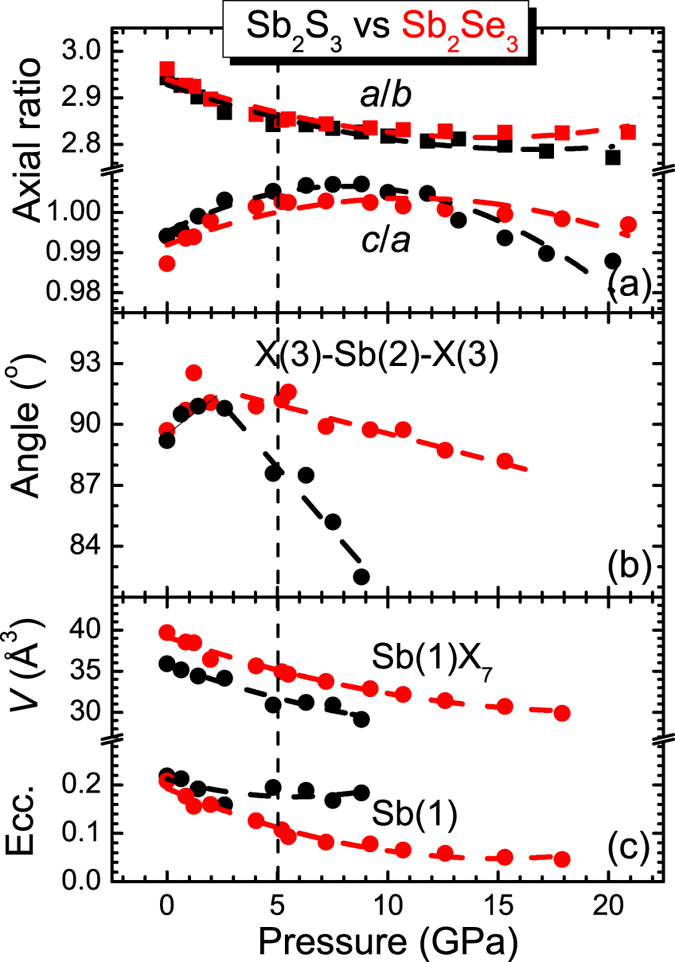
Structural comparison between Sb_2_S_3_ and Sb_2_Se_3_. Plot of (**a**) orthorhombic axial ratios, (**b**) the X(3)-Sb(2)-X(3) bond angle, (**c**) Sb(1)X_7_ polyhedral volumes, and the Sb(1) cation eccentricity as a function of pressure for both Sb_2_S_3_ (black) and Sb_2_Se_3_ (red, data from ref. 15). Dashed lines are guides to the eye. The vertical dashed line marks the isostructural transition.

Finally, we would like to briefly address the structural disorder in Sb_2_S_3_ initiating beyond 20 GPa (Fig. 3). Interestingly, the onset of this structural disorder lies at much lower pressures compared to Sb_2_Se_3_^15^ and Bi_2_S_3_^31^, but close with that of α-Sb_2_O_3_^44^. Such disorder can generally be accounted for by two mechanisms^45^: (a) the disordered phase may be a precursor of a structural transformation into another crystalline phase, which cannot be formed due to kinetic barriers, or (b) the tendency of the material to decompose into its constituents. Given the pressure-induced trends of Bi_2_Te_3_^19^,^35^,^46^, Sb_2_Te_3_^18^,^28^, Bi_2_Se_3_^20^,^37^,^47^,^48^,^49^, and Sb_2_Se_3_^15^ towards high-pressure disordered phases instead of elemental decomposition, however, the scenario of a kinetically-hindered structural transition in Sb_2_S_3_ appears more plausible. Actually, the appearance of the high-pressure modification above 15 GPa might be exactly that, i.e. the onset of a structural transformation of the *Pnma* phase towards another crystalline state, which is obstructed due to kinetic effects; a combined high-pressure and high-temperature structural study will be needed in order to resolve this matter. Considering nevertheless the structural trends of the A_2_B_3_ series under pressure, we can expect that this new structure will exhibit higher cationic coordinations.

In conclusion, our combined high-pressure Raman and XRD investigations revealed two phase transitions in Sb_2_S_3_ at 5 GPa and 15 GPa. The first transition is manifested via noticeable compressibility changes in several structural parameters. By taking into account an earlier report^16^, we assign these changes to a *second-order isostructural transition* arising from changes in the electronic structure of Sb_2_S_3_. Close comparison between the Sb_2_S_3_ and Sb_2_Se_3_ compounds up to 10 GPa reveals a slightly diverse pressure-induced behavior in the Sb^3+^ LEP activity after the isostructural transition, a plausible reason behind their different high-pressure electronic behavior above 5 GPa^12^,^16^. As for the second transition of Sb_2_S_3_ at 15 GPa, the new phase could not be identified due to the onset of structural disorder above 20 GPa. We speculate that the structural disorder is a transient state of this new high-pressure phase, which cannot be completed due to kinetic effects. Finally, an amorphous state is recovered upon full decompression from 53 GPa.

## Materials and Methods

### Sample and high-pressure technique details

Polycrystalline Sb_2_S_3_ powder was purchased commercially (Alfa-Aesar, 99.999% purity). The XRD measurements at ambient conditions did not detect any impurity phases. Pressure was generated with a gasketed symmetric diamond anvil cell, equipped with a set of diamonds with 300 μm culet diameter. The ruby luminescence method was employed for pressure calibration^50^.

### High-pressure Raman spectroscopy

The high-pressure Raman measurements were conducted with a solid-state laser (*λ* = 532 nm) coupled to a single-stage spectrometer and a charge-coupled device. The spectral resolution was 2 cm^−1^ and the lowest resolvable frequency was ~90 cm^−1^. Given the photo-sensitivity of the material^51^, the incident laser power was kept below 2 mW outside the DAC, whereas the size of the laser spot on the sample was approximately 30 μm. Mixtures of methanol-ethanol 4:1 and methanol-ethanol-water 16:3:1 served as pressure transmitiing media (PTM) in separate experimental runs.

### High-pressure angle-dispersive powder x-ray diffraction

The monochromatic angle-dispersive powder x-ray diffraction (XRD) measurements under pressure were performed at the 16BM-D beamline of the High Pressure Collaborative Access Team’s facility, at the Advanced Photon Source of Argonne National Laboratory (APS-ANL). The x-ray beam wavelength was *λ* = 0.4246 Å and the sample-detector distance about 320 mm. The XRD patterns were collected with a MAR345 Image Plate detector. The geometrical parameters were calibrated with a CeO_2_ standard from NIST. The intensity versus 2θ spectra were processed with the FIT2D software^52^. Refinements were performed with the GSAS + EXPGUI software packages^53^, whereas crystal-chemical calculations with the IVTON software^54^. The *P*-*V* data were fitted with a Birch-Murnaghan equation of state (B-M EoS)^55^. Helium was employed as PTM; the compressed gas loading took place at the gas-loading system of GeoSoilEnviroCARS^56^ (Sector 13, APS-ANL).

## Additional Information

**How to cite this article**: Efthimiopoulos, I. *et al*. Structural properties of Sb_2_S_3_ under pressure: evidence of an electronic topological transition. *Sci. Rep*. **6**, 24246; doi: 10.1038/srep24246 (2016).

## Supplementary Material

Supplementary Information

## Figures and Tables

**Table 1:** Mode assignment^23^, Raman mode frequencies, pressure coefficients, and the mode Gruneisen parameters γ of the Raman features of Sb_2_S_3_ calculated at a reference pressure *P*_R_.

Modesymmetry	*P*_R_(GPa)	*ω*_R_(cm^−1^)	∂*ω*/∂*P*(cm^−1^/GPa)	∂^2^*ω*/∂*P*^2^(cm^−1^/GPa^2^)	γ
A_g_(1)	10^−4^	100.4	0.6	−0.03	0.16
B_1g_(1)	10^−4^	108	2.2	−0.06	0.55
B_1g_(2)	10^−4^	127.5	3.5	−0.09	0.75
A_g_(2)	10^−4^	153.8	9.7	−0.4	1.72
A_g_(3)	10^−4^	190.8	3.6	−0.11	0.51
B_1g_(3)	10^−4^	202	2.6	–	0.35
B_1g_(4)	10^−4^	238	3.7	−0.07	0.42
A_g_(4)	10^−4^	281.5	−0.9	–	−0.09
	*5*	*271*	*3.1*	–	*0.74*
B_1g_(5)	10 − 4	300.1	−1.1	–	−0.1
	*5*	*295*	*−0.6*	–	*−0.13*
A_g_(5)		307	−0.8	–	0.07
**M1**	**15**	**127**	**1**	–	–
**M2**	**15**	**361**	**3.6**	–	–

The pressure depencence of the Raman-active modes is described by the relation: *ω*(*P*) = *ω*_R _+ α*P *+ b*P*^2^. Mode Gruneisen parameters γ are determined from the relation: γ = (*B*_0_/*ω*_R_) × (∂*ω*/∂*P*); the bulk modulus *B*_0_ = 27.2 GPa (or *B* = 65 GPa at 5 GPa) was employed.
