# Supplementary material for: Structural properties of Sb2S3 under pressure: evidence of an electronic topological transition
Source: Sci Rep. 2016 Apr 6;6:24246. doi: 10.1038/srep24246 (PMC4822153; doi:10.1038/srep24246)
Supplement: Supplementary Information [file srep24246-s1.pdf]

Structural properties of Sb_2S_3 under pressure: evidence of an electronic topological transition

Ilias Efthimiopoulos[1,*,†], Cienna Buchan[1], and Yuejian Wang[1,*]

[1]*Department of Physics, Oakland University, Rochester, MI, 48309, USA*

*Corresponding authors: iliefthi@gmail.com, ywang235@oakland.edu

†Current address: Helmholtz-Zentrum Potsdam, Deutsches GeoForschungsZentrum GFZ, Section 4.3, Telegrafenberg, 14473, Potsdam, Germany

Supplementary Information

Table S1: Lattice parameters for the *Pnma* phase of Sb_2S_3 at various pressures.

P (GPa)	*a* (Å)	*b* (Å)	*c* (Å)	*V* (Å^3)
10^{-4}	11.3045(2)	3.8411(1)	11.2386(2)	488.0
0.63	11.2014(2)	3.8268(1)	11.1525(2)	478.1
1.4	11.0658(2)	3.8127(1)	11.0555(2)	466.4
2.6	10.8969(2)	3.7977(1)	10.9316(2)	452.4
4.8	10.7277(2)	3.7731(1)	10.7843(2)	436.5
6.3	10.6986(2)	3.7636(1)	10.7708(2)	433.7
7.5	10.6316(2)	3.7502(1)	10.7065(2)	426.9
8.8	10.5625(2)	3.7366(1)	10.6373(2)	419.8
10	10.4935(3)	3.7235(1)	10.5467(3)	412.1
11.8	10.4068(3)	3.7067(1)	10.4566(3)	403.4
13.2	10.3897(3)	3.6946(1)	10.3704(3)	398.1
15.3	10.3069(3)	3.6823(1)	10.2419(3)	388.7
17.2	10.2412(3)	3.6756(2)	10.1365(3)	381.6
20.2	10.1479(4)	3.6606(2)	10.0245(4)	372.4

Table S2: Atomic coordinates for all ions in the Sb_2S_3 *Pnma* unit cell at various pressures.

P (GPa)	Sb(1)-x	Sb(1)-z	Sb(2)-x	Sb(2)-z	S(1)-x	S(1)-z	S(2)-x	S(2)-z	S(3)-x	S(3)-z
10^{-4}	0.0258(1)	0.6757(1)	0.3461(2)	0.4626(2)	0.0558(5)	0.1217(5)	0.3772(5)	0.0495(5)	0.2024(5)	0.7962(5)
0.63	0.0244(1)	0.6759(1)	0.3488(2)	0.4632(2)	0.0501(5)	0.1190(5)	0.3771(5)	0.0595(5)	0.2060(5)	0.8023(5)
1.4	0.0233(1)	0.6752(1)	0.3484(2)	0.4636(2)	0.0506(5)	0.1228(5)	0.3727(5)	0.0533(5)	0.2064(5)	0.8029(5)
2.6	0.0185(1)	0.6752(1)	0.3437(2)	0.4635(2)	0.0607(5)	0.1180(5)	0.3707(5)	0.0451(5)	0.2091(5)	0.8004(5)
4.8	0.0160(1)	0.6715(1)	0.3436(2)	0.4671(2)	0.0479(5)	0.1260(5)	0.3811(5)	0.0658(5)	0.2055(5)	0.7913(5)
6.3	0.0171(1)	0.6726(1)	0.3372(2)	0.4668(2)	0.0522(5)	0.1181(5)	0.3997(5)	0.0389(5)	0.2077(5)	0.7898(5)
7.5	0.0122(1)	0.6732(1)	0.3368(2)	0.4654(2)	0.0603(5)	0.1139(5)	0.3906(5)	0.0501(5)	0.2134(5)	0.7817(5)
8.8	0.0093(5)	0.6705(5)	0.3382(3)	0.4651(3)	0.073(1)	0.124(1)	0.390(1)	0.059(1)	0.208(1)	0.770(1)

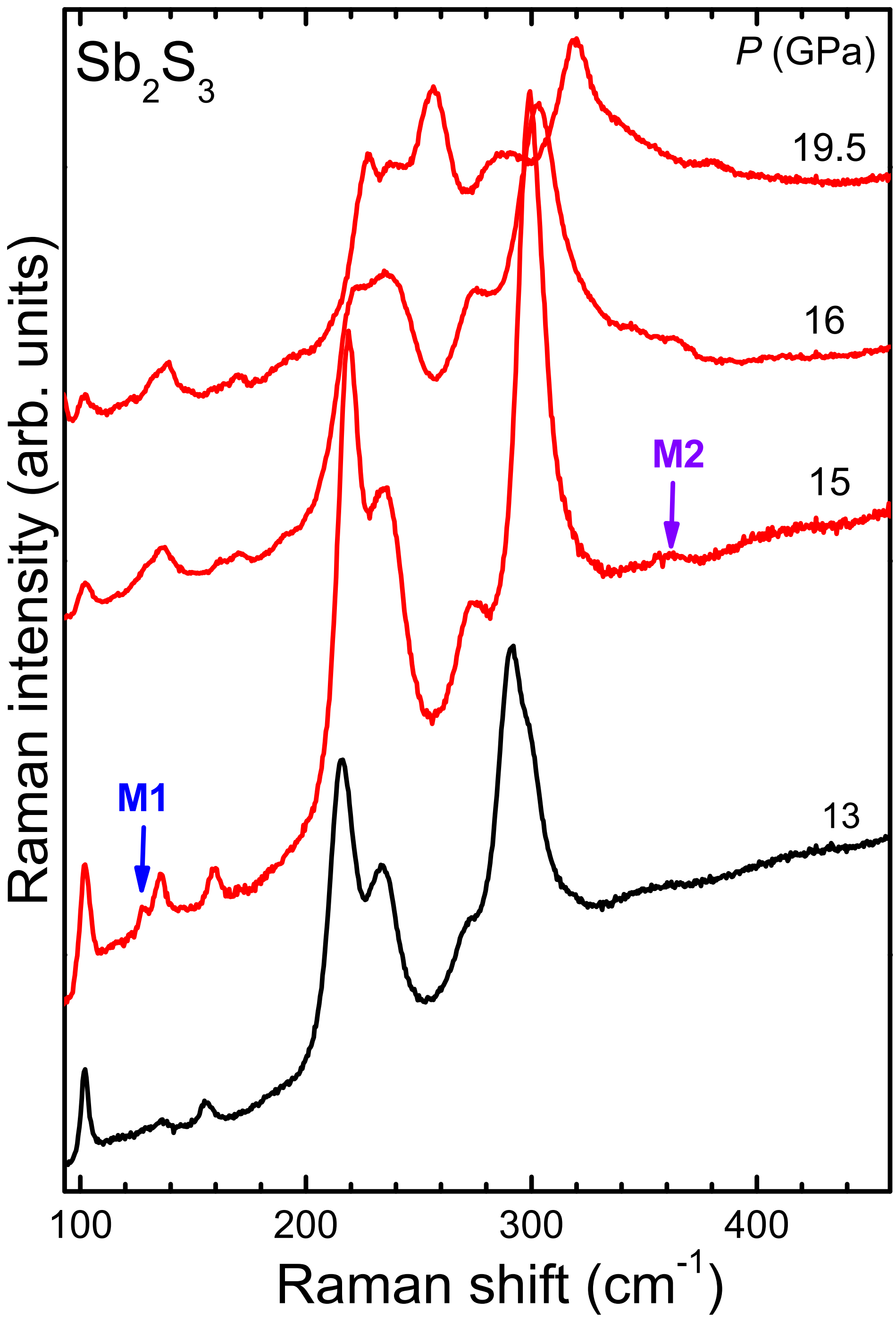

Figure S1: Raman spectra of Sb_2S_3 between 13-20 GPa (λ = 532 nm, T = 300 K). The "new" Raman features M1 and M2 are denoted with arrows.

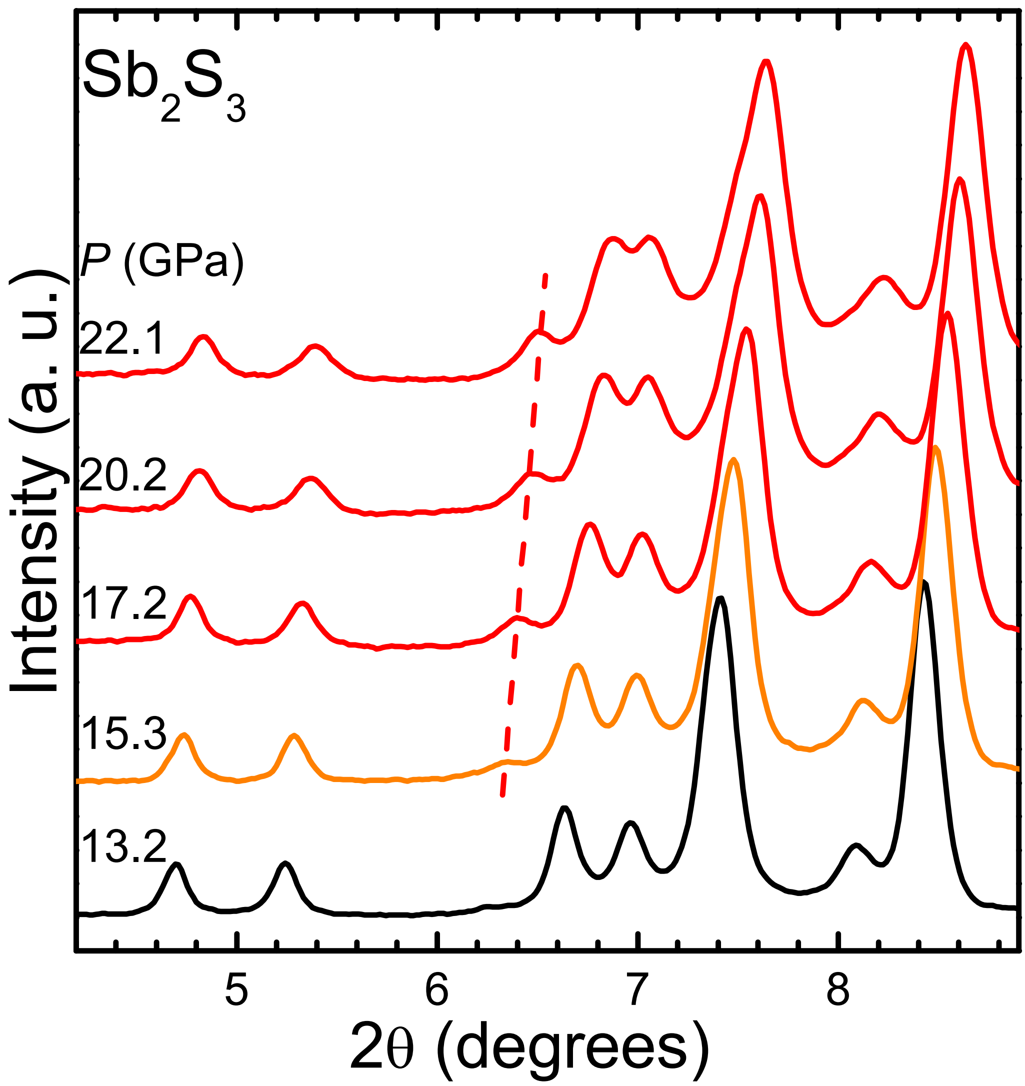

Figure S2: Expanded view of the XRD patterns of Sb_2S_3 between 13-22 GPa within the 4-9° 2θ range. The red dashed line marks the evolution of the new Bragg feature at 2θ ≈ 6.6^o.

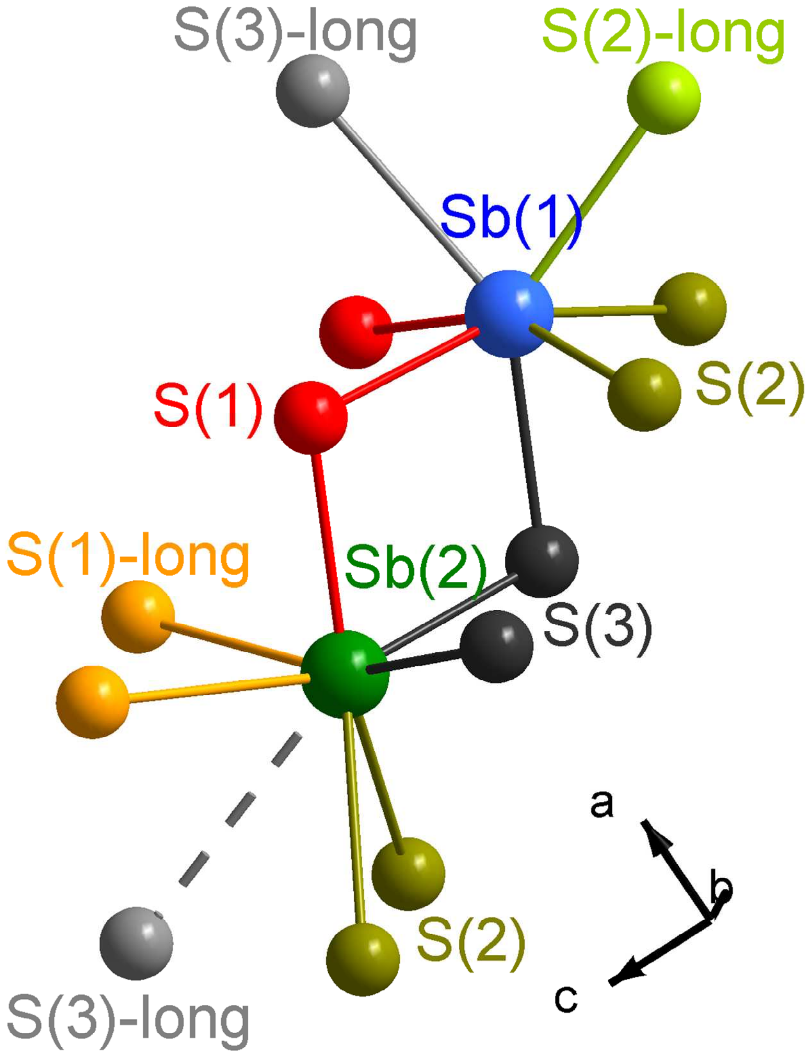

Figure S3: Sketch of the local coordination enviroment around the two distinct Sb(1) and Sb(2) ions of the Sb_2S_3 *Pnma* phase.
